# Childhood Environmental Exposures and Adult Disease

**DOI:** 10.1002/ppul.71591

**Published:** 2026-03-30

**Authors:** Andrew Bush

**Affiliations:** ^1^ National Heart and Lung Institute Imperial College, and Imperial Centre for Paediatrics and Child Health London UK; ^2^ Royal Brompton Hospital London UK

**Keywords:** aeroallergen, asthma, atopy, chronic obstructive pulmonary disease, lung cancer, nicotine, pollution, pulmonary fibrosis, viral infection

## Abstract

The association of early life adverse events with worse adult outcomes was first proposed by David Barker, the Developmental Origins of Health and Disease (DOHaD). Subsequently we have learned that adverse exposures are not merely important during pregnancy, but also exert transgenerational effects. Most of our knowledge derives from studies of nicotine and tobacco exposure. Adverse intrauterine exposures lead to low birth weight and premature delivery; altered airway and parenchymal structure; altered immune function; sensitization to later adverse exposures; and premature aging manifest by premature telomere shortening. Impaired spirometry for most tracks from birth to late middle age, and especially a low forced vital capacity, is associated with premature respiratory and all cause morbidity and mortality. Childhood exposure to passive smoking exacerbates antenatal effects on lung function, as does pollution. Pollution also increases the risk of early life respiratory infection which are a cause of impaired lung function in at least some studies, and also are associated with premature mortality. Diseases traditionally thought of as arising in adult life, including late‐onset and occupational asthma, chronic obstructive pulmonary disease, lung cancer and idiopathic pulmonary fibrosis all have their roots in early life adverse exposures, especially childhood onset smoking. Smoking provides proof of concept that prevention of adult disease must start earlier than adult life if it is to be effective. It is also likely that other early adverse exposures contribute to adult disease. Political action is needed to counteract the view that children “just grow out of it”. They do not.

## 1 Introduction

Professor David Barker promulgated the Developmental Origins of Health and Disease (DOHaD) hypothesis on the basis of seminal experiments in which he linked early life measurements such as birth weight and infant mortality from respiratory infection with adult outcomes such as death from chronic obstructive pulmonary disease [1, 2]. In essence, on the basis of large associative studies extending over decades, he proposed that “the origins of lifestyle‐related disease are formed at the time of fertilization, embryonic, foetal, and neonatal stages by the interrelation between genes and environmental influences such as nutrition, stress, and environmental chemicals”. Subsequent studies have shown that the early origins of disease are even earlier than he anticipated (below), but the principle, that adult diseases have their roots well before adult life, has repeatedly been confirmed. The relevant early adverse environmental exposures which potentially lead to adult disease will differ widely across the globe and even within countries; pockets of deprivation exist in high income settings and areas of extravagant wealth in low‐ and middle‐income countries. There are also marked rural‐urban differences [3]. The key exposure windows and relevant adverse exposures are summarised in Table 1. Most of the data on long term effects relates to tobacco exposure. There is a lot of data, not discussed here, on the short‐term effects of multiple adverse influences, but no direct evidence of a role in adult disease for want of appropriate long‐term studies. However, we can reasonably infer that if, for example, pollution exposure is associated with early impairment of lung function similar to tobacco, then as with tobacco exposure, COPD risk will be increased. When it comes to other comorbidities such as cardiovascular disease, it should not be assumed ahead of data that these are increased as they are by tobacco exposure.

**Table 1:** Key exposure windows.

Transgenerational: grandmother smoking affects her daughters' children; father active and passive smoking affects his offspring even if he quits long before their conception Pregnancy – indoor and outdoor pollution, maternal stress, maternal obesity, neighbourhood violence School years – indoor and outdoor pollution, respiratory infections, obesity

Perhaps the most dramatic demonstration of the effects of the environment on childhood disease, and by extension adult sequelae, comes from immigration studies. South Indian people living in South India have a very low prevalence of asthma. The prevalence of asthma who immigrated to Leicester after 4 years of age retained the low, South Indian risk of asthma, whereas those born in Leicester, or who settled there before their fourth birthday, had the high, Leicestershire asthma risk [4]. Another example comes from the Amish and Hutterite farming populations, genetically very similar, who fled to the USA from Europe to avoid persecution around 300 years ago. The Amish continued to use traditional farming methods, whereas the Hutterites switched to modern farms. The Amish have a far lower prevalence of atopy and asthma, associated with higher concentrations of endotoxin and greater microbial diversity in their environment [5], confirming many other studies on the importance of the early farming environment from across the world [6, 7]. A detailed discussion of possible mechanisms is beyond the scope of this manuscript, but it is undeniable that the early environment plays a key role in determining childhood disease. Indeed, it has been estimated that at least 40% of asthma can be prevented [8]. Furthermore, multiple overlapping cohort studies, summarised in [9], have shown that childhood asthma is associated with low lung function, manifest by either reduction in forced expired volume in 1 s (FEV_1_), increased rate of decline or premature onset of decline of FEV_1_. Importantly, both reduction in FEV1 and more rapid decline are both pathways to chronic obstructive pulmonary disease (COPD) (below). Childhood asthma is not something “you grow out of”.

This manuscript will focus on how adult disease is determined by childhood exposures without going into detail about the many different indoor, outdoor and other adverse effects which may impact normal early development. Details of the search strategy are in the Supporting Information. Since cardiovascular mortality and morbidity are intricately bound up with respiratory function this aspect will also be touched on briefly. The aim is to show that early adverse exposures should be considered not just a temporary inconvenience, but something that is associated with long term consequences not merely for the exposed child, but also for that child's subsequent children.

## 2 Before Barker – It Starts Earlier Than Was Thought?

An individual may be affected by adverse environmental factors long before conception [10, 11]. Two studies showed that if a grandmother smokes, her daughters are also more likely to smoke and have asthma. However, even if her daughter does not smoke, her own children (her mother's grandchildren) are more likely to have asthma. A third study confirmed this effect, but only in the offspring of women who did not smoke but whose mothers (the child's grandmother) did [12]. A recent epigenome wide association study documented abnormal cord blood DNA methylation in association with grandmaternal smoking, partially replicated in teenagers [13]. Paternal passive and active smoking is also associated with adverse effects, and two studies underscore that late pre‐puberty is a particularly vulnerable period. The Tasmanian cohort study which currently extends from the first to the sixth decade of life reported 1689 father‐offspring pairs. Passive smoke exposure in the father, before completing puberty, and later his active smoking increased the risk of non‐allergic childhood asthma in his offspring. The key risk window for passive exposure was below age fifteen years [14], a point relevant to other long‐term exposures (below). A second longitudinal, multi‐country study, reported on the exposure history of the parents of 24,168 offspring aged 2–51 years. Exposures included smoking (documenting the age at first taking up smoking), and occupational exposure to welding and metal fumes. The outcomes studied were a history of asthma and hay fever, including age at onset in the offspring. There was an increased risk of non‐allergic early‐onset asthma in the offspring of fathers but not mothers who smoked before conception, even if the father stopped smoking more than 5 years prior to conception. The same was seen with post‐pubertal welding exposure, and all findings were consistent across the seven Northern European countries studied [15]. Interestingly, there was no effect for females, and if anything, male adolescent smoking was worse than the mother smoking in her pregnancy.

The mechanisms of these associations are unclear. It may be that nicotine is the common factor, and if so, this would have implications for vaping. It is known that adolescent vaping causes widespread changes in airway gene expression, for example [16]. Whatever chemical(s) are involved these risks need to be widely known and taught to children before an age when they may start smoking, namely that their smoking may affect future children. It is likely that epigenetic pathways are involved, although this hypothesis is difficult to reconcile with the germline re‐programming which occurs at conception [17]. It is also unclear whether other adverse environmental exposures have transgenerational effects simply because there are no studies. However, what is clear is that developmental origins of adult disease may start way before conception.

### 2.1 Environmental Effects in Pregnancy: Very Far From Safe in the Womb

Most is known about the effects of tobacco and nicotine, and the consequences are summarised in Table 2. Although there is as yet no evidence of the effects of vaping in pregnancy, there is worrying animal data suggesting disruption of important developmental pathways in utero [18, 19, 20, 21, 22] and it would be a mistake to be complacent. The consequences of early smoking for adult disease will be discussed in subsequent sections.

**Table 2:** Consequences of adverse exposures, especially smoking, on the developing foetus.

Low birth weight Premature delivery Altered airway and parenchymal structure Altered immune function Sensitization to later adverse exposures Premature telomere shortening

### 2.2 Altered Airway and Parenchymal Structure

Much of the data has come from exposing pregnant animals to nicotine. To summarise, nicotine exposure leads to increased collagen deposition in the fetal lung [23], and increased MUC5AC and MUC5B gene expression [24]. There is failure of alveolar secondary septation leading to enlarged alveolar spaces and premature emphysema [25]. Airway growth is dysanaptic (longer than normal airways of normal calibre [26]; postnatally, dysanapsis is defined as a normal first second forced expired volume (FEV_1_), a greater than normal forced vital capacity (FVC) and thus a reduced FEV_1_/FVC ratio) [27]. In children with asthma, dysanapsis is associated with worse outcomes [28]. These anatomical changes also result in the newborn mice having airway hyper‐responsiveness (AHR), before exposure to infection and without any evidence of airway inflammation. Interestingly, three paediatric studies which measured AHR in the newborn period showed that this is a strong independent predictor of later asthma [29, 30, 31]. Autopsy studies in infants of mothers who smoked in pregnancy additionally have increased airway thickness [32] and loss of attachment points of the alveoli to the airway [33]. It is likely but unproven that pollution will have similar effects. Certainly, exposure of the pregnant mother to nitrogen dioxide and benzene was shown to impair spirometry at age 4.5 years in a dose‐dependent fashion in a study of more than 600 children [34]. Most if not all these children will have impaired lung function for life (see discussion of cohort studies below).

Altered immune function Cord blood studies taken at birth have shown that the babies of mothers who smoked in pregnancy have mononuclear cells that exhibit reduced Toll‐like receptor function [35]. There is an increased response blood mononuclear cell response to allergen exposure and altered cytokine responses correlating with future viral wheeze risk [36, 37]. Animal data confirms the adverse effects of antenatal tobacco smoke exposure on subsequent immune responses to infection [38]. The implications of early infections are discussed below.

### 2.3 Sensitization to Later Adverse Exposures

The consequences of these are discussed below, but there is no doubt that ante‐ and perinatal tobacco exposure primes the fetus to be worse affected by adult life exposures. Exposure of pregnant mice to tobacco smoke led to increased airway neutrophilia and delayed viral clearance during subsequent postnatal RSV infection in the pups [39]. In another study, perinatal or adult mice were exposed for 6 weeks to either filtered air or tobacco smoke [40]. They were then challenged intranasally with influenza virus or saline control. Neonatal mice which underwent either challenge showed increased inflammatory responses compared to control challenges perinatal or challenged adult mice. Antenatal and perinatal smoke exposure impairs host defence to subsequent viral infection.

### 2.4 Premature Telomere Shortening

Telomeres are found on the tips of chromosomes and shorten with age. Maternal smoking in pregnancy is associated with reduced cord blood telomere length [41, 42]. Other relevant environmental factors are deprivation, intrauterine growth retardation and bronchopulmonary dysplasia which are all associated with short telomeres aged 7–12 years [43]. At first sight maternal stress may not seem an environmental effect, but frequently it is related to poverty and deprivation, neighbourhood violence and other effects of low socio‐economic status. Maternal perceived stress in pregnancy was associated with reduced cord blood telomere length [44]. Maternal diet in pregnancy (often defined by poverty) is also associated with reduced cord blood telomere length [45]. Hence, adverse intrauterine exposures may leave the baby prematurely aged at birth. Childhood abuse is also associated with reduced telomere length [46].

### 2.5 Low Birth Weight

Birth weight is determined by many factors, including genetics and pre‐eclampsia, but cigarette smoking is a major factor. Low birth weight babies, even if not premature, have impaired lung function shortly after birth, and an increased risk of asthma in mid‐childhood [47]. The best data on the long‐term consequences of low birth weight are from David Barker's work. Low birth weight and weight at 1 year was associated with worse adult lung function and premature death from COPD. This was exacerbated by early childhood bronchitis, pertussis or pneumonia (another prescient finding anticipating modern studies, below) [1]. In another study, low birth weight was associated with respiratory and all‐cause mortality [48]. There are also important associations of low birth weight with cardiovascular disease, relevant for its association with lung function [49, 50, 51, 52], discussed in more detail below. Any adverse environmental effect leading to a low birth weight is likely to lead to long term respiratory and all‐cause consequences.

### 2.6 Premature Delivery

Although some babies are born prematurely as a result of an unforeseeable obstetric catastrophe such as placental abruption, there is no doubt that smoking is an important cause of prematurity. Preterm delivery is associated with long term airflow obstruction [53] and parenchymal changes [54] in adult life, even if the child required little or no respiratory support in the newborn period [55]. Pollution is also associated with worse the outcomes for babies with bronchopulmonary dysplasia (BPD) [56, 57]. Furthermore, it is clear that even early term delivery (37–39 weeks gestation) is associated with increased childhood respiratory morbidity [58]. Survivors of BPD are at risk of adult COPD [59]. However, whether the pathophysiology of chronic airflow limitation after prematurity is the same as COPD in adults with fixed airflow obstruction related to smoking or chronic asthma is not known.

### 2.7 Maternal Stress in Pregnancy

Maternal stress in pregnancy is associated with adverse effects on neonatal outcomes, but it may be difficult to tease out the exact factors responsible, because stress either antenatally or postnatally is rarely an isolated phenomenon [60]. Poverty and deprivation, pollution and neighbourhood violence are all potential confounders. Mediation may be direct or via the effects of premature delivery [61]. Maternal stressful events in pregnancy [62], as well as anxiety and depression [63] are all associated with an increased risk of childhood asthma. Mechanisms are unclear; results on whether the effects are mediated via atopy are conflicting [64, 65]. Epigenetic effects of stress have been described [66, 67]. We still have much to learn about mechanisms, but it is clear every effort should be made to reduce sources of stress such as poverty and neighbourhood violence.

### 2.8 The Early Years: They Do Not Grow Out of It

#### 2.8.1 The Importance of Understanding Trajectories

Key to understanding the importance of early life events is the normal and abnormal spirometric trajectories observed [68]. These have been clarified recently in a huge study combining 30,438 from eight different cohorts, born between 1901 and 2006. Just over half were female and the mean age was 26 years. There were 87,666 observations (range 2–8/participant). FEV_1_ was shown to have a biphasic rise in childhood, with an initial rapid rise until the early teenage years and then a slower rise to the peak, not plateau as previously thought, occurring at a mean age 20 years in females, and slightly later (mean age 23 years) in males. Forced vital capacity (FVC) showed a similar pattern. Decline from the peak was monophasic in both genders. A recent study demonstrated a tracking tool to follow the trajectory of an individual [69]. Numerous cohort studies of overlapping age ranges have demonstrated that most people track their growth centiles throughout life. However, there is also no doubt that a few people cross centiles upwards or downwards. In one study, early‐life risk factors were cumulatively associated with the very low lung function trajectory, as expected, and they were inversely associated with catch‐up [70]. Of practical importance, children with accelerated weight gain who subsequently lose weight exhibit catch‐up growth [60], as do children who do a lot of exercise [71]. For most, spirometry when you first walk through the school gates is the same as when you reach late middle age, and this fundamental principle must not be forgotten when considering the effects of early adverse exposures.

#### 2.8.2 Indoor and Outdoor Pollution

Passive and then active smoking act synergistically to impair lung function [72]. Household exposures, including chemicals and biomass fuels, which in particular differ across the globe, are another important factor [73]. The importance of air quality in schools has been increasingly stressed [74]. However, what is clear is that improving air quality results in improved lung growth. A study from California recruited three successive cohorts and followed them for 5 years. Each successive cohort breathed cleaner air as a result of legislation and had improved growth in FEV_1_ and FVC [75]. Also, although WHO have made recommendations about air quality, there really is no “safe” level of pollution [76]. Pediatricians must advocate for improving air quality for many reasons, but improving long‐term respiratory outcomes is one important one.

#### 2.8.3 Early Respiratory Infection

There is much evidence that indoor and outdoor pollution, and child poverty, all increase the risk of respiratory infections. The sinister implications of early respiratory tract infections is increasingly clear. A UK study in more than 5000 subjects demonstrated that respiratory infection in the first 2 years of life, and especially in the first year of life and especially if treated in hospital was associated with increased early mortality in adult life [77]. From these data it is unclear whether infection caused bad outcomes or was a marker of preceding adverse exposures leading both to bad outcomes and early infection. However, a study from a high‐infection‐burden community (Drakenstein, South Africa) showed that early respiratory infection was an independent risk factor for subsequent reduced lung function [78]. It should be noted that a study from Perth, Australia, showed by contrast that children admitted to hospital with bronchiolitis had premorbid impaired lung function, and this tracked into mid‐childhood [79]. Whether infection caused later impaired lung function or was merely a marker of risk driven by other factors, early infection is a marker of a high‐risk population, a fact that is missing from many childhood bronchiolitis and pneumonia guidelines.

#### 2.8.4 Allergen Exposure

The literature on the short‐ to medium‐term interactions between allergy and outcomes is extensive and will not be reviewed here. It should be noted that atopy is not an ‘all‐or‐none’ phenomenon, there being many different ‘atopies’ [80]. Of particular importance is early onset, multiple aeroallergen sensitisation, which is associated with worse asthma outcomes than other patterns of sensitisation. Multiple allergen sensitisation, early environmental tobacco smoke exposure, and acute attacks of wheeze together strongly predict progression from pre‐school wheeze to childhood asthma [81].

#### 2.8.5 Viruses and Allergy: The Effect on Asthma Attacks and Their Consequences

The combination of respiratory viral infection, allergen sensitisation and environmental allergen exposure is strongly predictive of asthma attacks [82]. These are more than just a temporary, albeit potentially severe, event, but are associated with progressive impairment of lung growth [83, 84], with the adverse consequences discussed below.

#### 2.8.6 Nutrition

Obesity is a disease of poverty. If you are poor, it is cheaper to fill the bellies of your children with junk food. A study of over 1200 children showed that, irrespective of birth weight, rapid weight gain in the first 2 years was associated with dysanapsis at age 7 years. Trajectories to obesity in adulthood start early in life [85]. David Barker's group showed that low birth weight and rapid weight gain was associated with premature death from coronary artery disease [86, 87]. A full discussion of the respiratory effects of maternal and child obesity is beyond the scope of this manuscript.

#### 2.8.7 Early Life Stress Including Neighbourhood Disadvantage

There are associations between violence and violence related stress, and asthma, although causality is unclear clear [88]. Associations with atopic [89] and TH17‐driven asthma [90] have been described, especially in marginalised youth communities. Low socioeconomic status (SES) associates directly and indirectly via violence with worse asthma morbidity [91]. As with pregnancy stress (above), teasing out the effects of confounders is difficult, but also as with pregnancy events, pediatricians must advocate for political action to reduce stressful triggers.

#### 2.8.8 More on Telomeres

Child abuse (physical, sexual. emotional) and neglect (physical and emotional), especially when multiple, is associated with premature telomere shortening [46], meaning premature aging of the individual.

#### 2.8.9 Genetic Effects

A detailed summary of genetic studies and lung function is beyond the scope of this article. One of the strongest gene associations with early onset asthma and viral infections is the 17q‐21 locus [92]. ADAM33 polymorphisms are also important predictors of early life lung function [93]. Preschool wheeze clinical phenotypes have distinct associations with genetic polymorphisms [94]. Genes associated with the risk of a preschool wheeze and asthma include Gasdermin B, Orosomucoid 1‐like 3, Cadherin‐related family member 3, Annexin A1, and IL33/IL1RL1. Epigenetic effects are important, especially methylation of cell‐type‐specific CpG sites. There are associations with airway‐remodelling and increased inflammatory responses, as well as enhanced susceptibility to environmental factors [95]. IL6 and CXCL10 were identified as potential markers of impaired lung development from childhood to adulthood [96]. Overall, although genetic factors are important, the environment is the chief driver of lung function trajectories.

### 2.9 Adult Life: Paying the Price

#### 2.9.1 COPD

This disease is characterised by airflow obstruction, lung parenchymal destruction (emphysema) and mucus hypersecretion (MUC5AC and MUC5B). The roots of all these features can be found antenatally (above). In addition, there is airway inflammation and infection. The Melbourne study was one of the first to demonstrate that the roots of COPD lie in early life, and with Tasmania (below) is one of two cohort studies spanning six decades (unfortunately, recruitment was not until the end of the first decade life, thus missing out the key very early time windows). The investigators recruited children with asthma and controls at age 7 years and enriched the cohort with children with severe asthma age ten years. When the cohort reached age 50, they re‐categorised the cohort into one of four groups, COPD, active asthma, remitted asthma, or no asthma. They showed that at the age of ten years the COPD group had far worse lung function than the others, and this tracked to age fifty [97]. Thus, the airway obstruction of COPD occurred in early life, and well before recruitment to the study. The Tasmanian and ECFS studies have also highlighted early the early childhood origins of COPD, particularly maternal smoking [98, 99]. A Danish longitudinal study highlighted the significance of early childhood “asthma‐like” respiratory symptoms with adult life COPD in more than 3000 children. In adult life, the symptomatic children were more likely to have impaired spirometry, hospital admissions for COPD and be prescribe a long‐acting muscarinic agent [100]. A seminal adult study showed that the two main routes to a diagnosis of COPD are failure to reach the normal peak of spirometry with a normal rate of decline (26% had a subsequent COPD diagnosis), and accelerated decline from a normal peak (7% had a subsequent COPD diagnosis [101]. The two tracks contributed equally to the population burden of COPD. Early life factors are clearly important in failure to reach the normal peak. Smoking was initially thought to be the cause of accelerated decline in lung function [102], but in a study of nearly 13,000 subjects, early life factors were shown to be important; maternal smoking, maternal age (for which I have no explanation) and winter birth (perhaps a marker for early life viral infections) were all associated with an accelerated rate of decline in lung function [103]. If the offspring later smoked as well, loss of lung function was even greater. Furthermore, there is no signal for active smoking as a cause of accelerated lung function decline in many large COPD cohorts. An important study highlighted again the increased vulnerability of the early pubertal period. In a study of more than 22,000 adults, a self‐reported COPD diagnosis was related to smoking history [104]. Those who started smoking before age 15 years had an approximate doubling of COPD risk irrespective of their level of smoking; they also had an increased lifetime exposure to tobacco and were less likely to quit smoking. The burden was highest in those of low socioeconomic status.

In summary, any environmental exposure that causes early airflow obstruction or increases the risk of early infection will likely contribute significantly to later COPD. It is likely the effects will be greater if they occur in the early prepubertal period, also supported by studies of lung cancer and idiopathic pulmonary fibrosis (IPF) below.

#### 2.9.2 Asthma

The roots of asthma are also early on. For example, the Tucson longitudinal study followed 180 infants through to age 36 years. They underwent infant pulmonary function at around 2 months of age, namely tidal breathing (tPTEF/tE) and rapid thoracoabdominal compression (V_max_ FRC). Those in the lowest tercile for either measurement had the highest prevalence of asthma at every time point. Two thirds of those in the lowest tercile for both had asthma in adult life. HRCT Those with low PTEF/tE had smaller, thinner airways on HRCT at age 26 years [105].

“Late‐onset” asthma is considered a diagnosis in adult life, being more prevalent in females. Also from Tucson, 1246 healthy newborns were prospectively followed to the third decade. Of those with physician‐diagnosed active asthma at 22 years of age, 25% (71% women) were newly diagnosed, typical “adult onset” disease. However, at age six these patients already had evidence of airway disease (wheeze, obstructive spirometry and bronchial hyper‐responsiveness). One lesson is that the recall of childhood events from the vantage of adult life is notoriously unreliable. Indeed, in one prospective study, adult recollection of child pertussis and pneumonia, both significant respiratory illnesses, was very unreliable. Both false positive and false negative recollections compared to prospectively acquired data were common [106].

#### 2.9.3 Occupational Asthma

Clearly, occupational asthma does not develop unless there is exposure to the allergen in question. However, early life factors also increase the risk of occupational asthma. In a study of more than 2000 office cleaners, maternal smoking, a severe respiratory tract infection in the first 5 years of life, being born in winter born and to a mother age more than 35 years increased the risk of wheeze, “adult‐onset” asthma and self‐reported COPD [107].

#### 2.9.4 Lung Cancer

A study based on the UK Biobank of nearly half a million subjects determined the effects of *in utero* smoke exposure and age of onset of smoking on lung cancer incidence and deaths during a median of nearly 9 years follow up. As expected, personal smoking was associated with both outcomes, but the earlier in life that smoking commenced, the greater the increased risk. In utero tobacco exposure also increased risk, and those who started smoking in childhood and had also been exposed in utero had a near eighteen‐fold increased lung cancer risk compared with non‐smokers. There was also an increased risk in those with an additional genetic predisposition [108].

#### 2.9.5 IPF

This disease also has roots in early life. Another UK Biobank‐based study reported 1134 incident IPF cases in nearly half a million subjects over a median follow‐up period of nearly eleven years [109]. The authors determined four risk factors. The first was a polygenic risk score. The second was phenoage (representing a composite of chronological age and nine biochemical markers). They also measured white cell telomere length and determined a lifetime smoking history. They sought interactions between the polygenic risk score, smoking history (maternal smoking in pregnancy and age at onset of personal smoking) and IPF, and the extent to which this was mediated by phenoage and telomere length. Irrespective of polygenic risk score and whether mother smoked, early age of smoking onset increased the IPF hazard ratio. Any history of smoking (maternal, child onset, adolescent onset, adult onset) was associated with increased IPF risk. Furthermore, older phenoage and shorter telomere length were both independently associated with increased IPF risk, accounting for c10% each. There was a 16‐fold higher risk for IPF in participants who had maternal smoke exposure, began smoking in childhood, and had a high polygenic risk score.

#### 2.9.6 CVS and Other Disease

Although a detailed discussion is beyond the scope of this manuscript, it should be noted that impaired spirometry, and especially a reduced FVC, is associated with premature cardiovascular and metabolic morbidity and mortality (James Hutchison who invented the spirometer did so because presciently he believed lung capacity was related to age at death, and used it to advise about life insurance policies). A study of nearly 7,500 people in the general population in the Atherosclerosis Risk in Communities (ARIC) dataset showed that survival was strongly associated with FVC after adjustment for FEV_1_, but not the other way round. There was no association between survival and airway obstruction (FEV_1_/FVC ratio [110]. The authors suggested we should be using the phrase “small lung syndrome” – in this context, small is certainly not beautiful, and other data have confirmed this. e.g., the Tasmanian cohort study which measured spirometry in nearly 2500 people between age 7 and 53 defined five FVC trajectories [111]. These were low FEV_1_/FVC only (obstructive), in 25.8%; low FVC only (restrictive), in 10.5%; both low FEV_1_/FVC ratio and low FVC (mixed), 3.5%; and normal trajectories, 60.2%. The mixed group had more respiratory disease, but only the restrictive pattern group had an increased risk of multimorbidity in middle age (including doctor‐diagnosed angina or myocardial infarction, hypertension, diabetes mellitus, and obstructive sleep apnoea).

The mechanism of these associations is unclear; it may partly be genetic. A genome‐wide association study (GWAS), involving over 150,00 people determined sixty loci where fetal genotype was associated with birth weight, itself a known risk factor for respiratory disease (above); around 15% of birth weight variance was accounted for by foetal genes [112]. There were strong inverse correlations between birth weight and systolic blood pressure, type two diabetes and coronary artery disease. Pathway analyses showed that genes within these sixty loci were enriched for insulin signalling, glucose homeostasis, glycogen biosynthesis and chromatin remodelling.

The UK Biobank studied variants in 55 lung development genes associated with adult FVC or FEV_1_/FVC, to see if there was any association with coronary heart disease, blood pressure, pulse pressure, Arterial Stiffness index and carotid intima‐ media thickness [113]. At least some replication was in the FinnGen and China Kadoorie Biobank studies. Twelve of the 55 genes shared the same variant between at least one lung function trait and at least one cardiovascular trait. A low FVC was the parameter which was most associated with cardiovascular risk. However, common environmental exposures, especially to tobacco, are likely also to play an important part.

### 2.10 The Next Generation: Paying the Price

Unfortunately, the effect of early adverse exposures crosses generations. A critical determinant of personal lung function is parental lung function, accounting for around 30% of the variance [114]. It is likely not widely appreciated that early life exposures have lifelong consequences and that these carry over into the next generation. Although most of the long term and transgenerational data are from studies of tobacco exposure, surely these are proof of concept, and it would be unwise to assume the same may not be true of other exposures. This makes it all the more imperative to break these cycles. Much could be done by political action, for example, tightening controls on smoking and vaping, tackling outdoor pollution and child poverty, and cracking down on junk food and other obesogenic diets. At an individual level, encouraging exercise and weight loss in the obese may improve lung function, and this can be tracked in the same way as with growth charts. Education is important; it is too late to start in the teenage years, but primary school children can easily understand that taking up smoking not merely has a permanent effect on their own health but may also affect their unborn children. It is not acceptable to ignore the present cycles of deprivation going from generation to generation.

## 3 Summary and Conclusions

Traditional “adult” diseases, such as COPD, late onset and occupational asthma, IPF and lung cancer in fact have their roots as early as antenatal and may also have transgenerational contributions. Most data on these early roots is from tobacco exposure. Many indoor and outdoor pollutants have been shown to have short‐ and medium‐term effects on for example, spirometry and risk of respiratory infection. We cannot confirm the long‐term significance of these without data, but the tobacco effects are not re‐assuring that these will be transient. Furthermore, small lungs are associated with extrapulmonary morbidity and mortality, and small lungs are likely to be transmitted to the next generation. Important knowledge gaps remain; we know very little about the mechanisms of how adverse effects translate into adverse biological outcomes. As has been stressed elsewhere [3], much can be achieved by political action, for example reducing smoking and vaping with heavy taxation, addressing environmental pollution and tackling childhood poverty and deprivation. Above all we must get across the message that if the major respiratory killers in adult life are to be prevented, efforts should start very early on, or no progress will be made.

## Author Contributions

I am the sole author and did everything.

## Conflicts of Interest

The author declares no conflicts of interest.

## 1 Data Availability Statement

Data sharing not applicable to this article as no datasets were generated or analysed during the current study.

## Supporting information

Supplementary material.
