# Supplementary material for: Childhood Environmental Exposures and Adult Disease
Source: Pediatr Pulmonol. 2026 Mar 30;61(4):e71591. doi: 10.1002/ppul.71591 (PMC13034404; doi:10.1002/ppul.71591)
Supplement: Supplementary file 1 — Supplementary material. [file PPUL-61-0-s001.docx]

**Supplementary material**

Pubmed was searched using a combination of <Environment> and <long term> and <consequences> limited to humans, children and English language. The search was supplemented by references from my personal archive, including from a recent book chapter [1]. The topic is huge, so the literature review cannot be comprehensive without writing multiple systematic reviews, and although I have tried to select the important papers, citation represents personal choice.

1. Sly PD, Bush A. Environmental contributions to respiratory disease in children. In: Bush A, Deterding R, Li A, Ratjen F, Sly P, Zar H, Wilmott R (Eds) Kendig and Wilmott’s disorders of the Respiratory Tract in Children, 10^th^ Edition. Publ Elsevier, Philadelphia, USA
